# Additive Manufactured Graphene Coating with Synergistic Photothermal and Superhydrophobic Effects for Bactericidal Applications

**DOI:** 10.1002/gch2.201900054

**Published:** 2019-10-07

**Authors:** Nan Jiang, Yilin Wang, Kang Cheung Chan, Ching‐Yuen Chan, Hongzhe Sun, Guijun Li

**Affiliations:** ^1^ Department of Chemistry The University of Hong Kong Hong Kong; ^2^ Advanced Manufacturing Technology Research Centre Department of Industrial and Systems Engineering The Hong Kong Polytechnic University Hong Kong; ^3^ State Key Laboratory of Ultra‐Precision Machining Technology Department of Industrial and Systems Engineering The Hong Kong Polytechnic University Hong Kong

**Keywords:** bacteriostasis, graphene, photothermal, robotics, superhydrophobic

## Abstract

Drug‐resistant bacterial infection is a global threat to public health due to the high mobility of the population. Novel therapy methods have been intensively studied for the eradication of antibiotic‐resistant bacteria, including photothermal treatment, which has established outstanding bacterial killing efficiencies under laser radiation, and superhydrophobic surfaces have exhibited excellent antifouling properties. However, an effective, scalable, and affordable bactericidal coating for eliminating drug‐resistant bacteria is lacking. Herein, a novel graphene coating using one‐step laser‐induced graphene and simultaneous laser‐induced forward transfer is introduced. The graphene coating shows high photothermal conversion and superhydrophobic performance, and these synergistic effects can make the bacteria number decrease with over 99.99% proportions under one sun illumination. The superhydrophobic properties can also reduce 99.87% of bacteria compared to the control sample when the solar energy is not available. This additive and scalable method can quickly coat functional graphene onto various substrates, with bacterial applications in many areas, such as water pipeline robots.

## Graphical abstract

A synergistic antibacterial graphene coating is developed for water pipeline robotics and has superhydrophobic and photothermal effects.

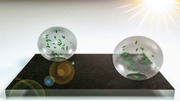

## 1 Introduction

Microbacterial infections are dangerous threats to public health.^1^ Sterilization is required in many places, such as hospitals, food industries, and fresh water transportation.^2^ Although chemotherapy methods have demonstrated effective bacterial killing performances over the past hundred years, the drug resistance due to the use of antibiotics has attracted increased attention in the recent years.^3^ A number of multidrug resistant bacterial, naming super bacteria, are causing great risks to public health.^4^ So alternative methods for antibacterial and bacterial‐killing are necessary.^5^

Photothermal therapy is a physical strategy for killing particular cells.^6^ Recent studies using near infrared (NIR) light for eradicating bacteria have confirmed the feasibility with various materials.^7^ Using plasmonic gold nanoparticles, outstanding antibacterial performance was verified under 808 nm laser irradiation.^8^ With 2D nanosheets, plasmonic heating, and silver release have also been demonstrated for killing both Gram negative *Escherichia coli* (*E. coli*) and Gram positive *Staphylococcus aureus* (*S. aureus*).^9^ The composition coating of gold nanoparticle and lysozyme film also showed over 99% killing rate for attached bacteria under laser irradiation.^10^ Semiconducting oligoelectrolytes have proven to have high photothermal efficiencies for killing Gram positive *Bacillus subtilis*.^11^ In vivo study of photothermal treatment using NIR also demonstrated efficient bacteria inhabitation, using nanorods decorated with glycomimetic polymers.^12^ Meanwhile, graphene materials also established low‐cost and efficient photothermal treatment.^13^ Multidrug resistant bacteria have been eradicated using polydopamine‐coated carboxyl graphene. Versatile antibacterial performance was also demonstrated using silver loaded graphene under NIR irradiation.^14^ Bacterial biofilms were successfully destroyed with capacity up to 70% using tobramycin conjugated graphene oxide.^15^ Thus, drug resistant bacteria can be efficiently sterilized using the photothermal effect under NIR laser irradiation.

However, the laser beam sizes are usually small and must be focused on the same spot for several minutes, limiting the wide use of this technology for sterilizing large areas. Solar energy is more abundant and can be used for larger area bacteria eradiation compared to NIR laser. Photothermal sterilization was also proven using Prussian blue nanocages under solar irradiation.^16^ Solar photocatalytic and photothermal effects have also been verified for effectively killing over 99.96% of bacteria under solar illumination.^17^ On the other hand, superhydrophobic antibacterial effects have also been validated using various materials.^18^ Herein, the most challengeable problem comes to be the combination of photothermal therapy with the antibacterial effects of superhydrophobicity and graphene materials, which can be inspired from the design of multifunctional nanomaterials acting as photothermal agents.^19^

Recently, we developed a novel and scalable method for the fabrication of large area graphene materials with superwetting and photothermal properties. Ultra‐high specific capacity supercapacitors have been fabricated using this laser induced forward transfer (LIFT) strategy.^20^ A superhydrophobic top layer can facilitate the self‐righting performance in Janus wetting films for solar steam generation.^21^ Laser patterned superhydrophilic graphene can simultaneously collect and detect the glucose level within sweat droplets.^22^ Herein, we further develop this LIFT strategy for an antibacterial coating of graphene. The synergistically photothermal and superhydrophobic effects have evidenced efficient bacterial killing toward Gram positive *E. coli*. This additive manufacturing method is scalable and affordable, and can be used in real‐world applications, such as antibacterial coatings for water pipeline robots.

## 2 Results and Discussion

**Figure 1:**
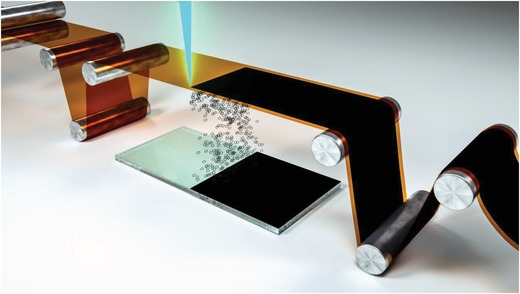
Schematic illustration of the process for coating graphene onto glass. A laser beam with 1064 nm wavelength was focused on the polyimide film. The synthesized graphene was forward transfer onto the glass substrate.

The LIFT deposition process is schematically illustrated as shown in **Figure** **1**. The laser beam is focused on a roll‐to‐roll type polyimide film, and the glass substrate is placed beneath the polyimide. When the laser beam is scanning according to the path designed by the computer software, the aromatic molecules within the polyimide can be photochemically reduced to graphene and forward transferred onto the glass substrates. This fully automatic process can be easily scaled up for coating objects with large sizes.

**Figure 2:**
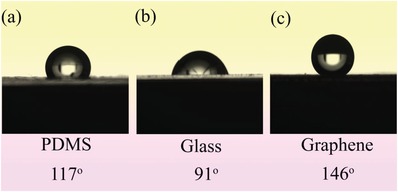
Water contact angle measurement of PDMS, glass, and glass substrate coating with graphene, respectively.

The surface energies of graphene are estimated using static contact angle measurement, in comparison with polydimethylsiloxane (PDMS) and glass substrates. The pristine PDMS substrate displayed hydrophobic behavior with a contact angle at 117°, as shown in **Figure** **2**a. The pristine glass substrate shows a less hydrophobic state with a contact angle at 91°, as depicted in Figure 2b. Meanwhile, the graphene coated glass substrate behaved as superhydrophobic, with a contact angle at 146°, as shown in Figure 2c. So the low surface energy can provide antibacterial properties, as described in the following sections.

**Figure 3:**
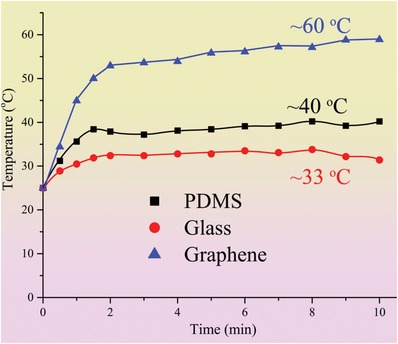
Relationship between temperature and solar illumination duration for different subtracts, including PDMS, glass, and graphene‐coated glass, respectively.

The photothermal effect of graphene compared to PDMS and glass substrates was explored, as shown in **Figure** **3**. The pristine glass substrate possesses high transmission rate for solar light and a relatively low stable temperature around 33 °C was achieved after 2 min of illumination, as shown as the blue curve in Figure 3. The pristine PDMS showed a slightly higher optical absorption compared to the pristine glass substrate. A higher temperature at 40 °C was stabilized after 2 min illumination on PDMS substrate under one sun intensity, as shown in the black curve in Figure 3. At the same time, the graphene coated glass showed the highest temperature under the same solar intensity at 1000 W m^−2^. The temperature increased up to 55 °C after the initial 2 min and stabilized around 60 °C after 10 min, as shown in the red curve in Figure 3. The above photothermal behavior proved that the graphene coated glass exhibited highest photon to thermo conversion rates.

**Figure 4:**
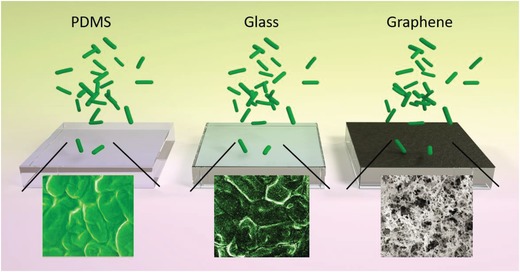
Schematic illustration of *E. coli* colonies on different substrates, including PDMS, glass, and graphene‐coated glass, respectively.

In combination with the outstanding superwetting and photothermal properties of such graphene, the synergistic antibacteria performance was examined. The *E. coli* colonies within agar plates were grown on pristine PDMS, pristine glass substrate, and graphene coated glass to examine the antibacteria effects, as illustrated in **Figure** **4**.

**Figure 5:**
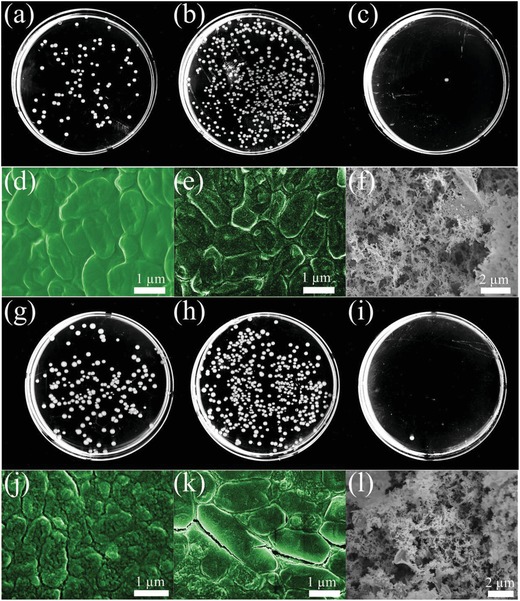
Characterization of *E. coli* colonies on different substrates without and with solar treatment. Optical image of *E. coli* colonies without solar treatment on a) PDMS, b) glass, and c) graphene‐coated glass. SEM image of *E. coli* colonies without solar treatment on d) PDMS, e) glass, and f) graphene‐coated glass. Optical image of *E. coli* colonies after 10 min solar treatment on g) PDMS, h) glass, and i) graphene‐coated glass. SEM image of *E. coli* colonies after 10 min solar treatment on j) PDMS, k) glass, and l) graphene‐coated glass.

**Figure 6:**
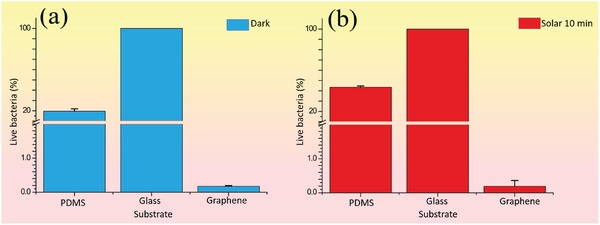
Normalized living rate of *E. coli* colonies on different substrates a) without solar treatment and b) with solar treatment. All the data were normalized by the number of colonies on glass with or without 10 min solar irradiation, respectively.

The *E. coli* colonies formed on the agar plates were examined with optical photos and electron microscopy. The optical images of the *E. coli* colonies on various substrates without solar treatment were characterized using 10^7^ dilution folds. Large numbers of *E. coli* colonies were observed in both the pristine PDMS (**Figure** **5**a) and glass substrates (Figure 5b). Fewer colonies were found for PDMS compared to glass, indicating that the more hydrophobic states of PDMS than glass can inhibit the *E. coli* growth. In contrast, only one colony was observed on the graphene coated glass substrate, as shown in Figure 5c. Statistically, the living bacteria colonies on graphene were 99.83% fewer than for pristine glass substrate, as depicted in **Figure** **6**a.

The microstructures of the *E. coli* on different substrates were examined using field emission scanning electron microscope (SEM). As shown in the field emission SEM, the dense biofilms of *E. coli* were clearly observed on the PDMS (Figure 5d) and glass substrates (Figure 5e) without solar treatment. The *E. coli* crowded against each other across the whole scanning area, with no significant differences for pristine PDMS and the glass substrate. However, there was no *E. coli* observed across the graphene coated glass area, with only porous graphene structures observed, as shown in Figure 5f.

After solar illumination for 10 min, significantly few colonies were observed in all samples. Only 100 folders of dilution were needed for examining the differences between various substrates. The living bacteria percentage difference on PDMS and glass substrate was reduced with solar treatment, compared to samples without solar treatment, as shown in Figure 5g,h. So it is indicated that the influence of photothermal effect is more significant than surface wetting properties for hydrophobic samples. At the same time, the graphene coated glass showed outstanding *E. coli* killing ability, leaving only 1 colony observed in Figure 5i. According to the colony counting, the graphene coated glass substrate showed higher bacteria decreasing rate (99.81%) than pristine glass under same solar illumination, as shown in Figure 6b. Compared to glass without solar illumination, the decreasing competence of the synergistic photothermal and superhydrophobic effect was around 99.99%.

Under electron microscopy, the photothermal antibacteria therapy was verified. More cracks on the biofilms were observed on the surface of pristine PDMS (Figure 5j) and glass substrates (Figure 5k) after 10 min solar irradiation. The *E. coli* were observed with very rough surfaces and physically separated from each other. So the strong photothermal effect can essentially influence the growth of *E. coli*. For the graphene coated glass substrate after the solar illumination, no *E. coli* was observed across multiple scanning areas, as shown in Figure 5l. The porous structure of graphene was well preserved, indicating the stability of such coating for working at this temperature. So the photothermal bacterial inhibition was successfully proven by the micro bacterial characterization.

With the synergistic photothermal and superhydrophobic effects, the graphene coatings have presented outstanding antibacterial performances using Gram negative *E. coli*. The photothermal effect of graphene under solar illumination can significantly inhibit the growth of *E. coli* by locally heating the surface to high temperature within minutes. At the same time, the low surface energy of the superhydrophobic graphene can prevent the fouling of biofilms. The integration of the two effects can effectively inhibit the micro bacteria growth.

This synergistic strategy shows significantly advances over existing reported research. Although NIR based photothermal antibacterial materials have already demonstrated outstanding performance in lab scale research, it is still challenging to apply this technique for large area application.^23^ The small laser beam has to be focused on the same position for minutes, and it takes quite a long time in scanning over large areas, spot by spot. At the same time, the reported solar photothermal lack the capability for low cost coating.^24^ In addition, these coatings lack superhydrophobic surface, leaving the surface easily fouled when solar energy is not present. Moreover, the sole superhydrophobic coating has shown some antibacterial effect, however, the bacterial killing rates were significantly lower than in current studies.^25^ So our synergistic photochemical and superhydrophobic effects can significantly increase the bactericidal performance.

**Figure 7:**
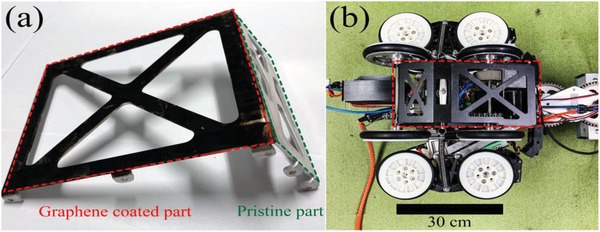
Photo of graphene coating on water pipe robot. a) The zoom‐in photo of the graphene coating part (in red dash frame), in comparison of the pristine metallic part (in green dash frame). b) The corresponding locations of such component on the water pipe robot.

This method can also be directly used for real‐world applications. For example, it can be used for coatings on plastic and metal components on the surface of water pipeline robots, as shown in **Figure** **7**. Before working within the water pipe, the 10 min duration of solar sterilization can make sure the robot would not pollute the pipeline.

## 3 Conclusions

In the present study, the bactericidal graphene coating was studied using robotic laser scribing. A superhydrophobic state is obtained using this material with a contact angle over 146°. When the coating is illuminated under one sun intensity, the surface temperature can be heated to 55 °C within 2 min and stabilized at 60 °C within 10 min. The synergistic superhydrophobic and photothermal effects can greatly reduce the bacterial colonies under solar illumination. Both superhydrophobic and photothermal properties of other functional materials, such as plasmonic nanoparticles and semiconducting materials, will be studied in the near future.

## 4 Experimental Section

Polyimide films from Shenzhen Zhengze company were used directly as raw materials without any treatment. A laser beam at 3 W power and 1064 nm wavelength was focused on the film for the LIFT deposition of graphene, using a scanning speed of 400 mm s^−1^. The glass substrates were cast coated with a thin layer of polydimethylsiloxane to enhance the surface adhesion of the deposited graphene onto glass. The morphologies of the *E. coli* on different substrates were characterized using a field emission scanning electron microscope, model Tescan MAIA3. The contact angles of different substrates were measured using a static session droplet machine, model Sindatek 100SB. A Newport solar simulator with an AM 1.5 filter was used for the solar illumination test.

The *E. coli* BL21 was incubated in Luria‐Bertani (LB) medium containing 50 mg L^−1^ kanamycin overnight at 37 °C before use. Then, these *E. coli* cells were diluted by PBS to prepare the suspensions with optical density at 600 nm (OD_600_) reaching 0.6. Then, 200 µL *E. coli* suspensions were used to be incubated on the surface of gelatinous LB agar plates (diameter = 10 cm) containing 1.5 wt% agar at 37 °C for 12 h. Ideally, *E. coli* cells can totally occupy all the surface. Samples (PDMS/Glass/Graphene) were then incubated with the *E. coli*‐occupied surfaces at 37 °C for 12 h in the dark whilst one more glass sample was set in each agar plate for the thickness control of *E. coli* cells. All the samples, except the thickness control, were subjected to 10 min solar treatment on white paper.

For SEM imaging, the above samples were soaked in liquid nitrogen prior to the following treatment. For colony counting assay, all the samples including the thickness control, were transferred to centrifuge tubes filled with 10 mL PBS. After 10 min shaking and 1 min sonication at 37 °C, the *E. coli* cells were detached from the samples and resuspended into PBS. After appropriately dilution by PBS, these *E. coli* suspensions were placed on gelatinous LB agar plates (diameter = 10 cm) containing 1.5 wt% agar at 37 °C for 18 h. Consequently, each living cell can develop into a distinct colony after incubation and the number of living *E. coli* cells was estimated by the visible colony numbers on the agar plates. All the experiments were performed independently twice, while the colony counting was operated in triplicate.

## Conflict of Interest

The authors declare no conflict of interest.

## Supporting information

Supplementary
