# Supplementary material for: Additive Manufactured Graphene Coating with Synergistic Photothermal and Superhydrophobic Effects for Bactericidal Applications
Source: Glob Chall. 2019 Oct 7;4(1):1900054. doi: 10.1002/gch2.201900054 (PMC6957018; doi:10.1002/gch2.201900054)
Supplement: Supplementary file 1 — Supplementary [file GCH2-4-1900054-s001.pdf]

Copyright WILEY-VCH Verlag GmbH & Co. KGaA, 69469 Weinheim, Germany, 2019.

Supporting Information

for *Global Challenges,* DOI: 10.1002/gch2.201900054

Additive Manufactured Graphene Coating with Synergistic Photothermal and Superhydrophobic Effects for Bactericidal Applications

Nan Jiang, Yilin Wang, Kang Cheung Chan, Ching-Yuen Chan, Hongzhe Sun, and Guijun Li**

Copyright WILEY-VCH Verlag GmbH & Co. KGaA, 69469 Weinheim, Germany, 2019.

Supporting Information

Additive Manufactured Graphene Coating with Synergistic Photothermal and Superhydrophobic Effects for Bactericidal Applications

Nan Jiang[1], Yilin Wang[2], Kang Cheung Chan[2], Ching-Yuen Chan[2], Hongzhe Sun[1] and Guijun Li*[2,3]*

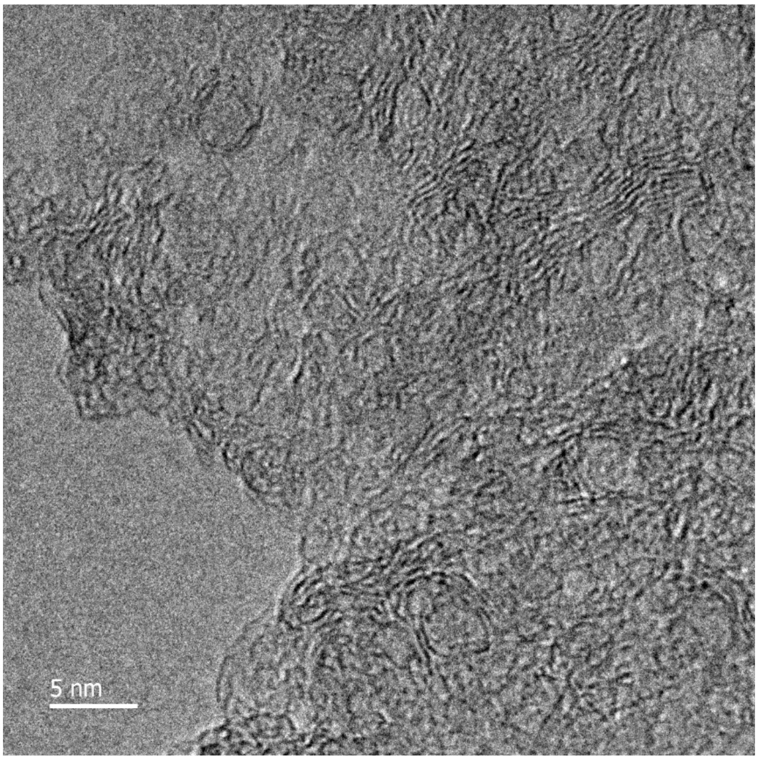

Figure S1. The TEM image of additive manufactured graphene coating.

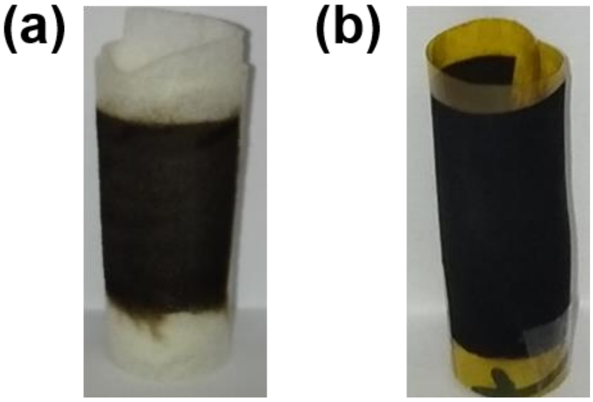

Figure S2. The good resistance of twisting demonstrated by the graphene coated substrates: (a) non-woven fabric and (b) polyimide.
